# A systematic evaluation of whole genome amplification of bisulfite-modified DNA

**DOI:** 10.1186/1868-7083-4-22

**Published:** 2012-11-22

**Authors:** Miki Bundo, Fumiko Sunaga, Junko Ueda, Kiyoto Kasai, Tadafumi Kato, Kazuya Iwamoto

**Affiliations:** 1 Department of Molecular Psychiatry, Graduate School of Medicine, The University of Tokyo, 7-3-1 Hongo, Bunkyo-ku, Tokyo, 113-8655, Japan; 2 Laboratory for Molecular Dynamics of Mental Disorders, RIKEN Brain Science Institute, 2-1 Hirosawa, Wako, Saitama, 351-0011, Japan; 3 Department of Neuropsychiatry, Graduate School of Medicine, The University of Tokyo, 7-3-1 Hongo, Bunkyo-ku, Tokyo, 113-8655, Japan

**Keywords:** DNA methylation, Sodium bisulfite modification, Cytosine modification

## Abstract

**Background:**

Studying DNA methylation profiles in detail should be the first step in epigenetic research. Although sodium bisulfite modification of genomic DNA is the gold standard method for DNA methylation analysis, this method results in the loss of the majority of the DNA material. Whole genome amplification (WGA) of bisulfite-modified DNA is expected to provide a rich source of materials, but its validity has not been thoroughly evaluated. In this study, we evaluated the extent of biased amplification in the WGA of bisulfite-modified DNA and the reproducibility of independent WGA reactions. We performed the multiple displacement amplification-based WGA separately three times. Each experiment included two reactions using 10 or 50 ng of bisulfite-modified DNA as template. DNA methylation levels were compared between WGA products and original bisulfite-modified DNA at about 450,000 CpG sites.

**Results:**

Using a sufficient amount of bisulfite-modified DNA for WGA was critical for downstream application. The considerable deviations from original bisulfite-modified DNA were found in the middle range of DNA methylation levels. Distribution of hyper- and hypomethylation were equal, which suggested that the deviation at each CpG site occurred randomly. Averaging the data from independently amplified WGA products dramatically improved the overall quality.

**Conclusions:**

WGA of bisulfite-modified DNA could be a valuable tool for epigenetic research, but careful experimental design and data interpretation are required.

## Background

In mammals, DNA methylation is mainly observed at the cytosine residues of CpG dinucleotides. The methyl group is transferred to the fifth position of cytosine by DNA methyltransferases. This modification plays important roles in the regulation of gene expression [1]. In the promoter region, where a CpG-rich region known as the CpG island is often situated, DNA methylation is generally involved in gene silencing. In the intragenic regions, where most of the methylcytosine is enriched, DNA methylation is associated with highly expressed genes and alternative splicing, although its precise role remains unclear [2-5].

DNA methylation is involved in genomic imprinting, X chromosome inactivation, and tissue-specific gene expression. Alteration of the DNA methylation results in developmental deficits and diseases [1,6]. Studies have shown that, in addition to cancer, various kinds of diseases, such as autoimmune diseases, diabetes, and neuropsychiatric diseases, are associated with altered DNA methylation [6-8]. Detailed qualitative and quantitative analyses of DNA methylation profiles should be the first step of epigenetic research in clinical medicine.

Sodium bisulfite modification of genomic DNA, which converts non-methyl cytosine to uracil, has been the gold standard method for DNA methylation analysis for decades. However, this method causes the degradation of genomic DNA, resulting in a loss of the majority of DNA material. Therefore, the amount of DNA required for such analyses is often on the order of micrograms.

Whole genome amplification (WGA) has been used to amplify genomic DNA for sequencing and genotyping analyses [9-11]. This method may also be valuable for epigenetic research, as WGA could provide a large amount of DNA. Because WGA products lose DNA methylation during amplification, bisulfite-modified DNA must be used as the template for WGA. One expected challenge is the unbiased amplification of bisulfite-modified genome DNA, which shows reduced genome complexity. In addition, unlike conventional genotyping, which is basically represented as categorical values (two homozygous alleles and one heterozygous allele), methylation levels are represented as continuous values ranging from 0 to 100%. It will be important, therefore, to develop a precise quantitative assay using WGA products, as well as a strategy for appropriate data interpretation.

Previous studies have reported on the validity and limitations of the application of WGA to bisulfite-modified genomic DNA [12-15], but only a handful of CpG sites were evaluated. Therefore, the extent of biased amplification in the WGA products and reproducibility of independent WGA reactions at the genome-wide level remain largely unclear, as does the effect of the quantity of starting materials. In this study, we systematically examined the validity and limitations of the WGA of bisulfite-modified genomic DNA.

## Results

### Whole genome amplification of bisulfite-modified DNA

**Figure 1:**
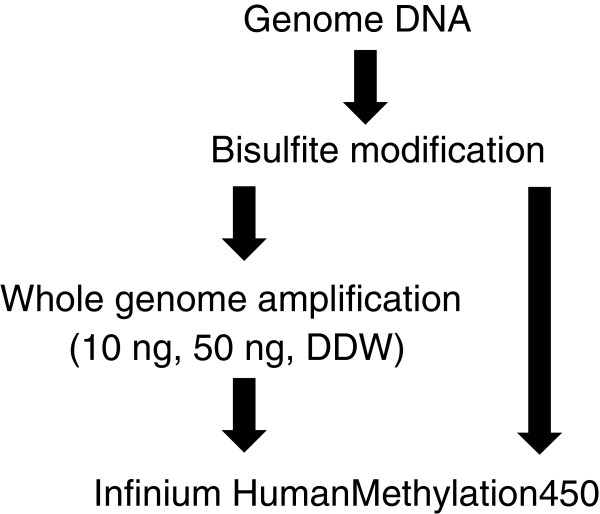
**Schematic diagram of the experimental design.** Genomic DNA extracted from peripheral blood was used for sodium bisulfite modification. Either 10 or 50 ng of bisulfite-modified DNA was used for multiple displacement amplification (MDA)-based whole genome amplification (WGA). We performed three independent WGA experiments. In each experiment set, deionized distilled water (DDW) was included as a negative control. Note that after bisulfite modification, DNA is in a single-stranded form.

We performed multiple displacement amplification (MDA)-based WGA by phi29 DNA polymerase using sodium bisulfite-modified DNA as the template. A schematic diagram of the experimental design is shown in Figure 1. To focus on the evaluation of WGA, bisulfite-modified DNA was prepared from genomic DNA derived from one subject, and all experiments were performed using an identical batch for bisulfite treatment. The WGA experiments were replicated three times. Each experiment includes three WGA reactions (10 or 50 ng of bisulfite-modified DNA as template, and deionized distilled water (DDW) as a negative control). Irrespective of the quantity of bisulfite-modified DNA, we obtained about 4 to 6 μg of amplified products (Table 1), whereas we did not observe significant amplification from negative controls. Agarose gel electrophoresis revealed no remarkable differences in product size across all WGA products (data not shown).

**Table 1:** **Yield and overall quality of whole genome amplification (WGA) products of bisulfite**-**modified DNA**

**Sample**	**WGA yield(ng)**	**Numberof the detected CpG**^a^	**Average methylation signal**	**Average unmethylation signal**
no amplification 1	-	484,857 (99.81 %)	1,764.202	2,537.532
no amplification 2	-	484,841 (99.81 %)	1,967.142	2,682.317
no amplification 3	-	484,831 (99.81 %)	1,882.296	2,875.583
WGA 50 ng 1	4,400	480,869 (98.99 %)	1,262.738	2,584.268
WGA 50 ng 2	6,000	480,159 (98.85 %)	1,266.639	2,113.009
WGA 50 ng 3	4,680	479,465 (98.70 %)	1,354.389	2,347.615
WGA 10 ng 1	4,300	465,730 (95.88 %)	1,208.001	2,257.573
WGA 10 ng 2	6,260	463,572 (95.43 %)	1,082.716	2,190.567
WGA 10 ng 3	6,020	467,036 (96.14 %)	1,292.537	1,931.764
DDW1	68	-	-	-
DDW2	58	-	-	-
DDW3	14	-	-	-

^a^Detection *P* value below 0.01 considered significant detection.

WGA, whole genome amplification; DDW, deionized distilled water.

### Genome-wide DNA methylation profiling of whole genome amplification products

We performed an Infinium HumanMethylation450 assay, which evaluates DNA methylation levels of about 450,000 CpG sites, using WGA products of bisulfite-modified DNA and original bisulfite-modified DNA. The total number of reliably detected CpG sites was significantly decreased after WGA, and depended on the quantity of bisulfite-modified DNA used for WGA reaction (Table 1). Average signal intensities of both methylation and unmethylation probes were also concordantly decreased in the WGA products (Table 1).

### Clustering and principal component analysis of whole genome amplification products

**Figure 2:**
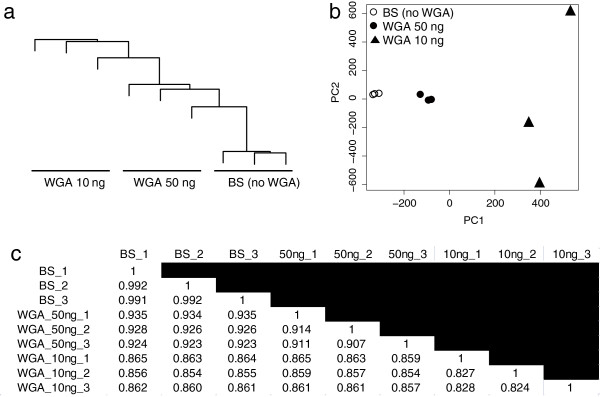
**Unbiased hierarchical clustering analysis(a),principal component analysis(b),and pairwise correlation analysis(c)of whole genome amplification (WGA) products of bisulfite**-**modified DNA.** The values indicate Spearman’s rho. BS, bisulfite-modified DNA.

Consistent with the lower number of detected CpG and decreased signal intensities in the WGA products, clustering analysis and principal component analysis clearly revealed that each WGA product of bisulfite-modified DNA showed a large deviation from original bisulfite-modified DNAs in a quantity-dependent manner (Figure 2a,b).

### Pairwise correlation

We calculated pairwise correlations of the methylation profile between all possible pairs (Figure 2c). Whereas correlations among original bisulfite-modified DNAs were very high (*R* = 0.991 to 0.992), Those correlations between original bisulfite-modified DNAs and WGA products decreased progressively according to the quantity of bisulfite-modified DNA, ranging from *R* = 0.923 to 0.935 for 50 ng and from *R* = 0.855 to 0.865 for 10 ng. Likewise, the average correlation among triplicates also decreased and was dependent upon the quantity of bisulfite-modified DNA (average *R* = 0.911 and 0.826 for 50 and 10 ng, respectively).

### Scatter plot analysis

**Figure 3:**
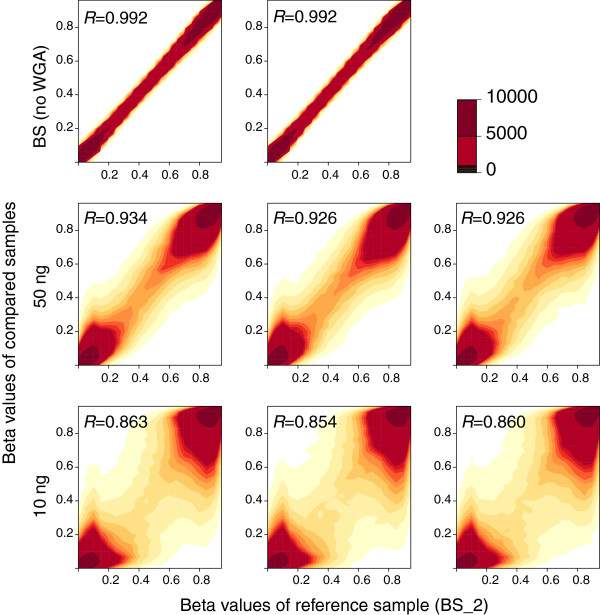
Scatter plot of beta values between reference sample and whole genome amplification (WGA) products.

**Figure 4:**
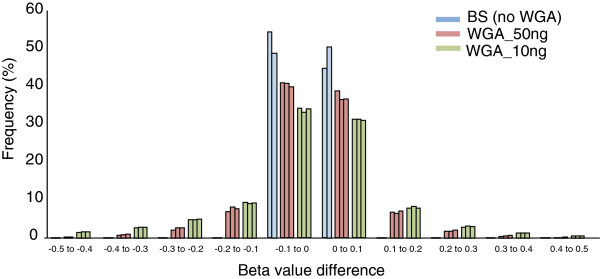
Distribution of beta value deviations from the reference sample.

We arbitrarily chose one original bisulfite modified DNA as a reference sample and calculated the correlation and beta value difference from those of other products (Figure 3). A beta value was calculated as the ratio of fluorescent signal intensity of the methylated probe to those of total (methylated and unmethylated) probes, and was considered as a DNA methylation level. The correlations were maintained at relatively high levels even in the WGA products using 10 ng of bisulfite-modified DNA (Figures 2 and 3). However, scatter plots clearly showed considerable deviations, especially in the middle range of beta values (from 0.3 to 0.7). A histogram of the beta value difference from the reference revealed that deviations in the WGA products were uniformly distributed in both directions (that is, hyper- and hypomethylation) (Figure 4). Whereas 99% of the total probes showed a beta value difference between 0.1 and −0.1 among the triplicates of original bisulfite-modified DNAs, only 78% and 65% of the total probe were included in this range for WGA products using 50 and 10 ng bisulfite-modified DNA, respectively.

### Effect of methylation levels

**Figure 5:**
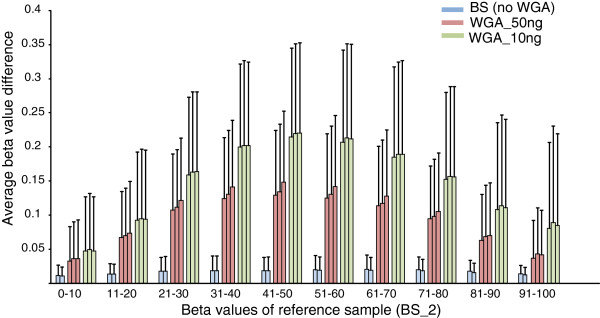
**Beta value differences with regard to methylation level.** The absolute beta value difference between whole genome amplification (WGA) product and the reference sample was calculated for each probe. Probes were divided into ten groups based on beta values of the reference sample. Values are mean and standard deviation.

We then examined the beta value difference with regard to methylation levels of the reference sample (Figure 5). In contrast to original bisulfite-modified DNAs, which showed high consistency over the entire range of beta values, the WGA products showed considerable deviations, especially in the middle range of beta values. At the level of 50% methylation, the average ± standard deviation beta value differences were 0.137 ± 0.100 and 0.218 ± 0.132 for WGA products of 50 and 10 ng of bisulfite-modified DNA, respectively.

### Validation of Illumina DNA methylation data by pyrosequencing

**Figure 6:**
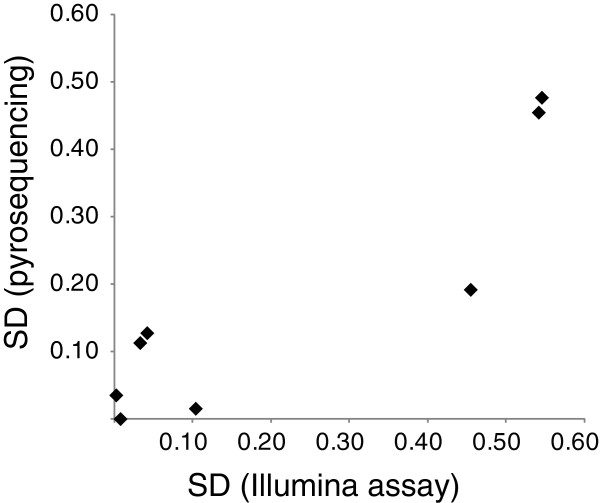
**Validation of extent of standard deviation by pyrosequencing.** A total of four CpG sites (see Table S1 in Additional file 1 for detail) were selected for analysis. In each CpG site, DNA methylation levels were measured by pyrosequencing at two different amounts of input DNA (50 ng and 10 ng) in triplicate. Standard deviations determined by Illumina assay and pyrosequencing were plotted.

We arbitrary chose the CpG sites showing various degrees of standard deviation (SD) of beta values after WGA (see Table S1 in Additional file 1). We then determined DNA methylation levels at each CpG site using pyrosequencing. The correlation between Illumina assay and pyrosequencing was very high (*R* = 0.921). Standard deviations determined by pyrosequencing showed consistent results with those determined by the Illumina assay (*R* = 0.900) (Figure 6).

### Effect of averaging the multiple WGA products

**Figure 7:**
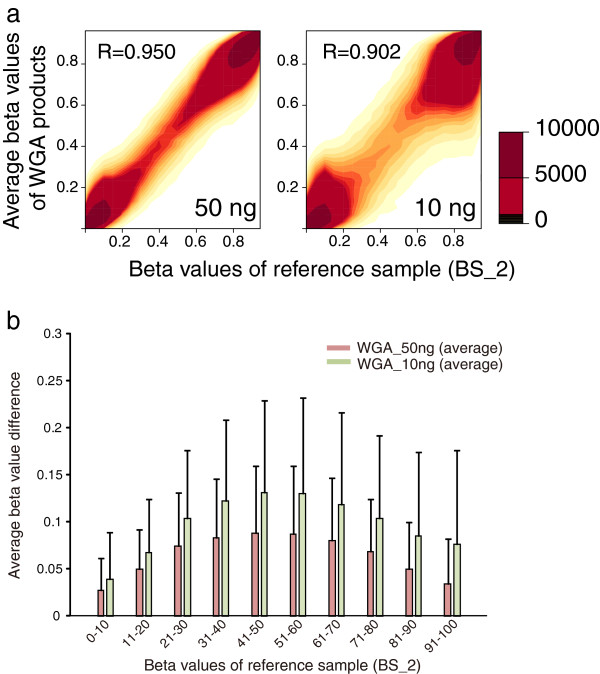
**Effect of averaging independently analyzed data of whole genome amplification (WGA) products.** (**a**) Scatter plot of beta values between the reference sample and averaged WGA products. (**b**) Beta value differences of averaged WGA products. The absolute beta value difference between average WGA product and the reference sample was calculated for each probe. Values are mean and standard deviation.

Given that amplification biases occurred randomly (Figure 4), we expected that averaging multiple WGA products would reduce the deviations. In fact, averaging the triplicate experimental data considerably improved amplification biases in WGA products of both 50 ng (*R* = 0.950) and 10 ng (*R* = 0.902) of bisulfite-modified DNA (Figure 7a). Consistency of the middle-range methylation levels was also markedly improved. At the level of 50% methylation, the average ± standard deviation beta value differences were 0.088 ± 0.071 and 0.131 ± 0.097 for WGA products of 50 and 10 ng of bisulfite-modified DNA, respectively (Figure 7b).

## Discussion

We examined the characteristics of MDA-based WGA of bisulfite-modified genomic DNA in detail. First, our analyses revealed that when performing WGA, using a sufficient amount of bisulfite-modified genome DNA is critical. Comparison between WGA products using 50 or 10 ng of bisulfite-modified DNA clearly showed more deviations in the 10 ng reactions. Second, although methylation levels were relatively conserved and showed little deviation in the hypomethylated (beta value <0.3) and hypermethylated (>0.7) regions, considerable deviations were found in the middle range of DNA methylation levels. These findings were not platform-dependent results, as the independent pyrosequencing analysis confirmed the extent of deviations (Figure 6). This result is consistent with previous examinations of several representative CpG sites [12-14]. Third, given the nature of random amplification deviations, averaging the multiple WGA products considerably reduces the deviation. Averaging three WGA products of 10 ng of bisulfite-modified DNA showed better results with regard to beta value difference in the middle range of methylation levels as compared with WGA product of 50 ng of bisulfite-modified DNA (Figure 7).

In terms of clinical settings, researchers must be cautious when using WGA-based products for the detection of subtle methylation differences, which are often required for case–control studies of common diseases. There are several requirements for such an application: (1) the expected methylation difference should be relatively large between groups; (2) the use of a sufficient amount of bisulfite-modified DNA for WGA; and (3) preparation of independent WGA replicates whenever possible. Practically, pooling the independent WGA reactions is expected to be effective to reduce the deviations. One alternative approach would be to treat the methylation level as a categorical variable. For example, when we categorized methylation levels of the reference sample as hypomethylation (beta value <0.3) and hypermethylation (beta value >0.7), 94% and 92% of the hypomethylated and 92% and 83% of the hypermethylated probes were correctly detected in WGA product of 50 and 10 ng of bisulfite-modified DNA, respectively (data not shown).

In this study, we employed the MDA-based WGA method. Alternative WGA would be the primer extension preamplification (PEP)-based method [16]. As PEP involves PCR reaction by DNA polymerase, MDA is believed to produce more unbiased amplified products [17]. However, a previous study reported that both methods provided comparable results when bisulfite-modified DNA was used as template [13]. One major drawback of MDA-based method would be the insufficient amplification from severely degraded DNA template [11]. As bisulfite modification causes DNA degradation, low amount of input DNA will be resulted in the failure of unbiased amplification. Therefore, improving the bisulfite modification method, which prevents high degradation of DNA template, would be one of useful steps for further reduction of input DNA.

## Conclusions

WGA of bisulfite-modified DNA may serve as a valuable tool for epigenetics research in the clinical medicine, although careful experimental design and data interpretation will be required.

## Methods

### DNA sample

This study was approved by the ethics committee at the University of Tokyo Hospital. Genomic DNA was extracted from peripheral blood of an adult Japanese female by using a standard phenol-chloroform extraction. Quality and integrity was assessed by optical density (OD) measurement and gel electrophoresis. Quantity of genomic DNA was measured using a Qubit dsDNA BR assay kit (Life Technologies, Carlsbad, Califolnia, USA) with a Qubit fluorometer (Life Technologies).

### Bisulfite modification and purification

Sodium bisulfite modification of genomic DNA was performed with an EpiTect Bisulfite kit (Qiagen, Venlo, Netherlands), according to the manufacturer’s instructions. The quantity of bisulfite-modified DNA was measured using Qubit ssDNA assay kit (Life Technologies) with a Qubit fluorometer.

### Whole genome amplification of bisulfite-modified DNA

WGA of bisulfite-modified DNA was performed using EpiTect Whole Bisulfitome kit (Qiagen), according to the manufacturer’s instructions. In brief, bisulfite-modified DNA was amplified with a reaction buffer containing phi29 DNA polymerase at 28 °C for 8 h. WGA included three independent experiments. In each experiment, the sample set contained 50 or 10 ng of bisulfite-modified DNA and DDW as the negative control. In performing WGA, UV-irradiation of all the equipment successfully suppressed nonspecific amplification from negative controls [18] (data not shown).

### Illumina Infinium assay

An Illumina Infinium HumanMethylation450 assay (Illumina) was performed according to the manufacturer’s protocol. The DNA methylation level was represented as a beta value, which was calculated as the ratio of fluorescent signal intensity of the methylated probe to those of total (methylated and unmethylated) probes by the GenomeStudio software (Illumina) with the default settings. All data are publicly available (GSE39565).

### Data analysis

We used the detection *P* value, which estimates the confidence that the signals from the target CpG probe are above those from the negative control probes, and average signal intensities for methylation and unmethylation probes were used to compensate for the quality of WGA products. *P* values below 0.01 were considered to represent specific detection of the target CpG probes. Spearman’s correlation and principal component analysis were performed with R (ver. 2.13.0; http://www.R-project.org/). Hierarchical clustering analysis was performed by another multidimensional analysis package (amap) implemented in the R software package [19].

### Pyrosequencing

DNA methylation levels of the selected CpG sites were measured by PSQ 96MA (Qiagen), according to the manufacturer’s instructions. Briefly, bisulfite-PCR product using a biotin labeled primer was mixed with a binding buffer containing streptavidin-sepharose beads. The reaction mixture was placed onto a MultiScreen-HV, Clear Plate (Millipore, Billerica, Massachusetts, USA). After applying the vacuum, the beads were treated with a denaturation solution, and were suspended with an annealing buffer containing a sequencing primer. The mixture was transferred onto a PyroMark Q96 Plate Low (Qiagen). Sequencing reaction was performed with a PyroMark Gold Q96 Reagents Kit (Qiagen). The percentage of methylation was calculated using the allele quantification algorithm of the PyroMark Q96 ID software 2.5.8.15 (Qiagen). Primers were listed in Table S1 in Additional file 1. Detailed methods are available upon request.

## Abbreviations

WGA: Whole genome amplification; MDA: Multiple displacement amplification; DDW: Deionized distilled water.

## Competing interests

M.B. F.S. and K.I. belong to the Department of Molecular Psychiatry, which is an endowment department by Dainippon Sumitomo Pharma, Yoshitomi Yakuhin, and Astellas Pharma. These companies had no role in study design, data collection and analysis, decision to publish, or preparation of the manuscript.

## Authors’ contributions

M.B. and F.S. performed the experiments. M.B., J.U., and K.I. analyzed the data. T.K., K.K. and K.I. supervised the study. M.B. and K.I. wrote the manuscript. All authors read and approved the final manuscript.

## Supplementary Material

Additional file 1 **Table S1.** Primers used in this study. (XLSX 9 kb) Click here for file
